# Supplementary material for: Aggressiveness as a latent personality trait of domestic dogs: Testing local independence and measurement invariance
Source: PLoS One. 2017 Aug 30;12(8):e0183595. doi: 10.1371/journal.pone.0183595 (PMC5576744; doi:10.1371/journal.pone.0183595)
Supplement: S1 Text — (PDF) [file pone.0183595.s001.pdf]

S1 Text. Obtaining model predictions for the graphical posterior predictive checks

In Figures 1 and 2, we obtained the predicted probabilities of aggression in each context and sex and/or age group by marginalising over the random effects with the approximation given by Zeger et al. (1988: 1054):

$$\frac{\beta x}{(1 + c^2 + \sigma^2)}$$

where β is the regression coefficient for some explanatory variable x, c is $16\sqrt{3}/(15\pi)$ and σ^2 is the variance of the random effect (e.g. random intercept). Applying this approximation allowed for the model predictions to be comparable to the probabilities of aggression in the raw data. In hierarchical logistic regression models, the estimates conditional on the random effects (i.e. conditional on the random intercepts being at their mean, in this case zero) can be larger in absolute value than the marginal estimates. In our case, the conditional estimates were lower than the marginal estimates (since estimates on the log-odd scale were negative), meaning that the model predicted probabilities in Figures 1 and 2 are slightly higher than their corresponding conditional estimates. The conditional estimates were used for plotting purposes only, and without any loss of information, to provide graphical posterior predictive checks against the raw data. The numerical results reported in text are all conditional estimates.

References

Zeger SL, Liang KY, Albert PS. Models for longitudinal data: a generalized estimating equation approach. Biometrics. 1988, Dec 1:1049-60.
