# Supplementary material for: Aggressiveness as a latent personality trait of domestic dogs: Testing local independence and measurement invariance
Source: PLoS One. 2017 Aug 30;12(8):e0183595. doi: 10.1371/journal.pone.0183595 (PMC5576744; doi:10.1371/journal.pone.0183595)
Supplement: S1 Table — The number of dogs who had 0, 1, and > 1 observations of aggression while at the shelter. (PDF) [file pone.0183595.s002.pdf]

S1 Table. Counts of aggression per context. The number of dogs who had 0, 1, and > 1 observations of aggression while at the shelter.

**Context**	**0 (n)**	**1 (n)**	**> 1 (n)**
Handling	0 (n = 4286)	1 (n = 338)	> 1 (n = 103)
In kennel towards people	0 (n = 3990)	1 (n = 340)	> 1 (n = 332)
In kennel towards dogs	0 (n = 4050)	1 (n = 365)	> 1 (n = 219)
Interactions with familiar people	0 (n = 4217)	1 (n = 291)	> 1 (n = 63)
Interactions with unfamiliar people	0 (n = 4104)	1 (n = 421)	> 1 (n = 181)
Out of kennel towards people	0 (n = 4491)	1 (n = 165)	> 1 (n = 29)
Out of kennel towards dogs	0 (n = 3855)	1 (n = 455)	> 1 (n = 375)
Interactions with food	0 (n = 4279)	1 (n = 255)	> 1 (n = 45)
Interactions with toys	0 (n = 4348)	1 (n = 125)	> 1 (n = 18)
Interactions with female dogs	0 (n = 3097)	1 (n = 623)	> 1 (n = 168)
Interactions with male dogs	0 (n = 2923)	1 (n = 753)	> 1 (n = 265)
