# Supplementary material for: Aggressiveness as a latent personality trait of domestic dogs: Testing local independence and measurement invariance
Source: PLoS One. 2017 Aug 30;12(8):e0183595. doi: 10.1371/journal.pone.0183595 (PMC5576744; doi:10.1371/journal.pone.0183595)
Supplement: S2 Table — Tetrachoric correlations between aggression contexts on the raw binary data, before the multiple imputation. Abbreviations used: HND (Handling); FPL (Interactions with familiar people); UPL (Interactions with unfamiliar people); KD (In kennel towards dogs); KP (In kennel towards people); OKD (Out of kennel towards dogs); OKP (Out of kennel towards people); EAT (Eating food); TOY (Interactions with toys); DM (Interactions with male dogs); DF (Interactions with female dogs). (PDF) [file pone.0183595.s003.pdf]

S2 Table. Tetrachoric correlations between aggression contexts. Tetrachoric correlations between aggression contexts on the raw binary data, before the multiple imputation. Abbreviations used: HND (*Handling*); FPL (*Interactions with familiar people*); UPL (*Interactions with unfamiliar people*); KD (*In kennel towards dogs*); KP (*In kennel towards people*); OKD (*Out of kennel towards dogs*); OKP (*Out of kennel towards people*); EAT (*Eating food*); TOY (*Interactions with toys*); DM (*Interactions with male dogs*); DF (*Interactions with female dogs*).

	HND	FPL	UPL	KD	KP	OKD	OKP	EAT	TOY	DM	DF
**HND**	1	0.585	0.446	0.046	0.396	0.108	0.346	0.499	0.448	0.134	0.179
**FPL**	0.585	1	0.568	0.051	0.526	0.007	0.413	0.471	0.39	0.136	0.121
**UPL**	0.446	0.568	1	0.089	0.746	0.134	0.607	0.387	0.242	0.093	0.12
**KD**	0.046	0.051	0.089	1	0.153	0.337	0.135	0.008	-0.002	0.422	0.447
**KP**	0.396	0.526	0.746	0.153	1	0.136	0.531	0.403	0.139	0.131	0.154
**OKD**	0.108	0.007	0.134	0.337	0.136	1	0.176	0.095	0.143	0.315	0.285
**OKP**	0.346	0.413	0.607	0.135	0.531	0.176	1	0.272	0.244	0.122	0.192
**EAT**	0.499	0.471	0.387	0.008	0.403	0.095	0.272	1	0.59	0.116	0.130
**TOY**	0.448	0.39	0.242	-0.002	0.139	0.143	0.244	0.590	1	0.12	0.058
**DM**	0.134	0.136	0.093	0.422	0.131	0.315	0.122	0.116	0.120	1	0.540
**DF**	0.179	0.121	0.120	0.447	0.154	0.285	0.192	0.130	0.058	0.540	1
