# Supplementary material for: Aggressiveness as a latent personality trait of domestic dogs: Testing local independence and measurement invariance
Source: PLoS One. 2017 Aug 30;12(8):e0183595. doi: 10.1371/journal.pone.0183595 (PMC5576744; doi:10.1371/journal.pone.0183595)
Supplement: S3 Table — Mean and 95% highest density interval (HDI) estimates for all parameters from the Bayesian hierarchical logistic model assessing measurement invariance for contexts reflecting aggressiveness towards people. Differences between levels of categorical variables are indicated by ‘.v.’ in the parameter name; interactions are denoted with ‘*’ in the parameter name. The decision rule for each parameter is given except for those variables not interpreted inferentially: YES = 95% HDI falls completely outside the region of practical equivalence (ROPE); NULL = 95% HDI falls completely inside the ROPE; ROPE = 95% HDI partly covers the ROPE. (PDF) [file pone.0183595.s004.pdf]

S3 Table. Bayesian hierarchical model parameter estimates for aggression towards people in different contexts. Mean and 95% highest density interval (HDI) estimates for all parameters from the Bayesian hierarchical logistic model assessing measurement invariance for contexts reflecting aggressiveness towards people. Differences between levels of categorical variables are indicated by '.v.' in the parameter name; interactions are denoted with '*' in the parameter name. The decision rule for each parameter is given except for those variables not interpreted inferentially: YES = 95% HDI falls completely outside the region of practical equivalence (ROPE); NULL = 95% HDI falls completely inside the ROPE; ROPE = 95% HDI partly covers the ROPE.

Parameter	Mean	HDI low	HDI high	Decision
Weight	0.928	0.863	0.987	Not interpreted
Total days	1.863	1.745	1.99	Not interpreted
London centre .v. Brands Hatch centre	1.7	1.525	1.895	Not interpreted
London centre .v. Old Windsor centre	2.026	1.849	2.204	Not interpreted
Brands Hatch centre.v. Old Windsor centre	0.841	0.74	0.947	Not interpreted
Not neutered .v. neutered on site	2.418	2.205	2.631	Not interpreted
Not neutered .v. neutered	1.649	1.506	1.793	Not interpreted
Neutered .v. neutered on site	1.468	1.35	1.6	Not interpreted
Gift.v.Return	0.707	0.608	0.808	Not interpreted
Gift.v.Stray	2.471	2.273	2.678	Not interpreted
Stray.v.return	0.286	0.242	0.328	Not interpreted
Females.v.males	0.719	0.668	0.77	YES
Handling.v.In kennel towards people	0.535	0.494	0.576	YES
Handling.v.Out of kennel towards people	2.821	2.536	3.078	YES
Handling.v.Eating food	1.51	1.379	1.644	YES
Handling.v.Interactions with toys	3.45	3.089	3.806	YES
Handling.V.Interactions with familiar people	1.314	1.203	1.432	ROPE
Handling.V.Interactions with unfamiliar people	0.622	0.58	0.675	YES
In kennel towards people.V.Out of kennel towards people	5.273	4.786	5.771	YES
In kennel towards people.V.Eating food	1.51	1.379	1.644	YES
In kennel towards people.V.Interactions with toys	6.45	5.823	7.122	YES
In kennel towards people.V.Interactions with familiar people	2.456	2.261	2.656	YES
In kennel towards people.V.Interactions with unfamiliar people	1.163	1.078	1.243	NULL
Out of kennel towards people.V.Eating food	0.536	0.484	0.592	YES
Out of kennel towards people.V.Interactions with toys	1.225	1.086	1.366	ROPE
Out of kennel towards people.V.Interactions with familiar people	0.466	0.42	0.512	YES
Out of kennel towards people.V.Interactions with unfamiliar people	0.221	0.201	0.241	YES
Eating food.V.Interactions with toys	2.287	2.038	2.532	YES
Eating food.V.Interactions with familiar people	0.871	0.789	0.948	ROPE

Eating food.V.Interactions with unfamiliar people	0.412	0.378	0.447	YES
Interactions with toys.V.Interactions with familiar people	0.382	0.344	0.426	YES
Interactions with toys.V.Interactions with unfamiliar people	0.181	0.163	0.2	YES
Interactions with familiar people.V.Interactions with unfamiliar people	0.474	0.435	0.512	YES
4 to 10 months.v.10 months to 3 years	0.638	0.565	0.705	YES
4 to 10 months.v.3 to 6 years	0.744	0.654	0.828	ROPE
4 to 10 months.v.Over 6 years	0.842	0.736	0.952	ROPE
10 months to 3 years.v.3 to 6 years	1.166	1.069	1.271	ROPE
10 months to 3 years.v.Over 6 years	1.32	1.188	1.453	ROPE
3 to 6 years.v.Over 6 years	1.133	1.013	1.261	ROPE
Handling.V.In kennel towards people*female.v.male	0.779	0.67	0.895	ROPE
Handling.V.Out of kennel towards people*female.v.male	0.578	0.481	0.682	YES
Handling.V.Eating food*female.v.male	1.042	0.88	1.222	NULL
Handling.V.Interactions with toys*female.v.male	0.787	0.636	0.933	ROPE
Handling.V.Interactions with familiar people*female.v.male	1.033	0.87	1.193	NULL
Handling.V.Interactions with unfamiliar people*female.v.male	0.616	0.53	0.702	YES
In kennel towards people.V.Out of kennel towards people*female.v.male	0.743	0.623	0.867	ROPE
In kennel towards people.V.Eating food*female.v.male	1.339	1.145	1.559	ROPE
In kennel towards people.V.Interactions with toys*female.v.male	1.012	0.83	1.197	NULL
In kennel towards people.V.Interactions with familiar people*female.v.male	1.329	1.136	1.522	ROPE
In kennel towards people.V.Interactions with unfamiliar people*female.v.male	0.792	0.701	0.902	ROPE
Out of kennel towards people.V.Eating food*female.v.male	1.812	1.495	2.152	YES
Out of kennel towards people.V.Interactions with toys*female.v.male	1.369	1.066	1.652	ROPE
Out of kennel towards people.V.Interactions with familiar people*female.v.male	1.798	1.488	2.126	YES
Out of kennel towards people.V.Interactions with unfamiliar people*female.v.male	1.072	0.891	1.243	NULL
Eating food.V.Interactions with toys*female.v.male	0.759	0.609	0.904	ROPE
Eating food.V.Interactions with familiar people*female.v.male	0.996	0.836	1.174	NULL
Eating food.V.Interactions with unfamiliar people*female.v.male	0.594	0.506	0.686	YES
Interactions with toys.V.Interactions with familiar people*female.v.male	1.321	1.088	1.595	ROPE
Interactions with toys.V.Interactions with unfamiliar people*female.v.male	0.788	0.651	0.929	ROPE
Interactions with familiar people.V.Interactions with unfamiliar people*female.v.male	0.598	0.513	0.687	YES
Handling.V.In kennel towards people*4 to 10 months.v.10 months to 3 years	1.353	1.071	1.651	ROPE
Handling.V.Out of kennel towards people*4 to 10 months.v.10 months to 3 years	0.958	0.722	1.228	ROPE
Handling.V.Eating food*4 to 10 months.v.10 months to 3 years	0.706	0.533	0.884	ROPE
Handling.V.Interactions with toys*4 to 10 months.v.10 months to 3	0.882	0.622	1.147	ROPE

years				
Handling.V.Interactions with familiar people*4 to 10 months.v.10 months to 3 years	1.048	0.798	1.292	ROPE
Handling.V.Interactions with unfamiliar people*4 to 10 months.v.10 months to 3 years	1.204	0.992	1.413	ROPE
In kennel towards people.V.Out of kennel towards people*4 to 10 months.v.10 months to 3 years	0.712	0.527	0.883	ROPE
In kennel towards people.V.Eating food*4 to 10 months.v.10 months to 3 years	0.524	0.4	0.642	YES
In kennel towards people.V.Interactions with toys*4 to 10 months.v.10 months to 3 years	0.655	0.483	0.851	ROPE
In kennel towards people.V.Interactions with familiar people*4 to 10 months.v.10 months to 3 years	0.779	0.613	0.951	ROPE
In kennel towards people.V.Interactions with unfamiliar people*4 to 10 months.v.10 months to 3 years	0.895	0.754	1.037	ROPE
Out of kennel towards people.V.Eating food*4 to 10 months.v.10 months to 3 years	0.744	0.553	0.964	ROPE
Out of kennel towards people.V.Interactions with toys*4 to 10 months.v.10 months to 3 years	0.93	0.641	1.227	ROPE
Out of kennel towards people.V.Interactions with familiar people*4 to 10 months.v.10 months to 3 years	1.105	0.823	1.416	ROPE
Out of kennel towards people.V.Interactions with unfamiliar people*4 to 10 months.v.10 months to 3 years	1.27	1.025	1.547	ROPE
Eating food.V.Interactions with toys*4 to 10 months.v.10 months to 3 years	1.26	0.864	1.643	ROPE
Eating food.V.Interactions with familiar people*4 to 10 months.v.10 months to 3 years	1.498	1.153	1.928	ROPE
Eating food.V.Interactions with unfamiliar people*4 to 10 months.v.10 months to 3 years	1.721	1.403	2.059	YES
Interactions with toys.V.Interactions with familiar people*4 to 10 months.v.10 months to 3 years	1.208	0.87	1.594	ROPE
Interactions with toys.V.Interactions with unfamiliar people*4 to 10 months.v.10 months to 3 years	1.387	1.035	1.72	ROPE
Interactions with familiar people.V.Interactions with unfamiliar people*4 to 10 months.v.10 months to 3 years	1.158	0.951	1.364	ROPE
Handling.V.In kennel towards people*4 to 10 months.v.3 to 6 years	1.074	0.841	1.325	ROPE
Handling.V.Out of kennel towards people*4 to 10 months.v.3 to 6 years	0.501	0.365	0.645	YES
Handling.V.Eating food*4 to 10 months.v.3 to 6 years	0.861	0.657	1.084	ROPE
Handling.V.Interactions with toys*4 to 10 months.v.3 to 6 years	0.598	0.428	0.799	YES
Handling.V.Interactions with familiar people*4 to 10 months.v.3 to 6 years	0.733	0.555	0.914	ROPE
Handling.V.Interactions with unfamiliar people*4 to 10 months.v.3 to 6 years	1.015	0.825	1.201	NULL
In kennel towards people.V.Out of kennel towards people*4 to 10 months.v.3 to 6 years	0.47	0.355	0.606	YES
In kennel towards people.V.Eating food*4 to 10 months.v.3 to 6 years	0.806	0.636	1.021	ROPE
In kennel towards people.V.Interactions with toys*4 to 10 months.v.3 to 6 years	0.56	0.405	0.731	YES
In kennel towards people.V.Interactions with familiar people*4 to 10 months.v.3 to 6 years	0.686	0.533	0.852	ROPE
In kennel towards people.V.Interactions with unfamiliar people*4 to 10 months.v.3 to 6 years	0.951	0.788	1.104	ROPE
Out of kennel towards people.V.Eating food*4 to 10 months.v.3 to 6 years	1.74	1.233	2.234	ROPE
Out of kennel towards people.V.Interactions with toys*4 to 10	1.208	0.839	1.624	ROPE

months.v.3 to 6 years

Out of kennel towards people.V.Interactions with familiar people*4 to 10 months.v.3 to 6 years	1.48	1.079	1.915	ROPE
Out of kennel towards people.V.Interactions with unfamiliar people*4 to 10 months.v.3 to 6 years	2.051	1.608	2.543	YES
Eating food.V.Interactions with toys*4 to 10 months.v.3 to 6 years	0.701	0.487	0.926	ROPE
Eating food.V.Interactions with familiar people*4 to 10 months.v.3 to 6 years	0.859	0.637	1.085	ROPE
Eating food.V.Interactions with unfamiliar people*4 to 10 months.v.3 to 6 years	1.19	0.969	1.429	ROPE
Interactions with toys.V.Interactions with familiar people*4 to 10 months.v.3 to 6 years	1.247	0.85	1.645	ROPE
Interactions with toys.V.Interactions with unfamiliar people*4 to 10 months.v.3 to 6 years	1.726	1.302	2.159	YES
Interactions with familiar people.V.Interactions with unfamiliar people*4 to 10 months.v.3 to 6 years	1.398	1.135	1.673	ROPE
Handling.V.In kennel towards people*4 to 10 months.v.Over 6 years	0.382	0.292	0.485	YES
Handling.V.Out of kennel towards people*4 to 10 months.v.Over 6 years	0.278	0.198	0.373	YES
Handling.V.Eating food*4 to 10 months.v.Over 6 years	0.777	0.576	1	ROPE
Handling.V.Interactions with toys*4 to 10 months.v.Over 6 years	0.822	0.578	1.111	ROPE
Handling.V.Interactions with familiar people*4 to 10 months.v.Over 6 years	0.435	0.323	0.554	YES
Handling.V.Interactions with unfamiliar people*4 to 10 months.v.Over 6 years	0.367	0.297	0.447	YES
In kennel towards people.V.Out of kennel towards people*4 to 10 months.v.Over 6 years	0.733	0.502	0.96	ROPE
In kennel towards people.V.Eating food*4 to 10 months.v.Over 6 years	2.048	1.539	2.606	YES
In kennel towards people.V.Interactions with toys*4 to 10 months.v.Over 6 years	2.164	1.516	2.83	YES
In kennel towards people.V.Interactions with familiar people*4 to 10 months.v.Over 6 years	1.147	0.858	1.46	ROPE
In kennel towards people.V.Interactions with unfamiliar people*4 to 10 months.v.Over 6 years	0.966	0.786	1.164	ROPE
Out of kennel towards people.V.Eating food*4 to 10 months.v.Over 6 years	2.847	1.92	3.777	YES
Out of kennel towards people.V.Interactions with toys*4 to 10 months.v.Over 6 years	3.01	1.908	4.113	YES
Out of kennel towards people.V.Interactions with familiar people*4 to 10 months.v.Over 6 years	1.594	1.117	2.148	ROPE
Out of kennel towards people.V.Interactions with unfamiliar people*4 to 10 months.v.Over 6 years	1.342	0.99	1.705	ROPE
Eating food.V.Interactions with toys*4 to 10 months.v.Over 6 years	1.068	0.729	1.421	ROPE
Eating food.V.Interactions with familiar people*4 to 10 months.v.Over 6 years	0.566	0.408	0.731	YES
Eating food.V.Interactions with unfamiliar people*4 to 10 months.v.Over 6 years	0.476	0.381	0.585	YES
Interactions with toys.V.Interactions with familiar people*4 to 10 months.v.Over 6 years	0.54	0.377	0.727	YES
Interactions with toys.V.Interactions with unfamiliar people*4 to 10 months.v.Over 6 years	0.454	0.34	0.581	YES
Interactions with familiar people.V.Interactions with unfamiliar people*4 to 10 months.v.Over 6 years	0.851	0.668	1.035	ROPE
Handling.V.In kennel towards people*10 months to 3 years.v.3 to 6 years	0.796	0.672	0.928	ROPE

Handling.V.Out of kennel towards people*10 months to 3 years.v.3 to 6 years	0.526	0.409	0.631	YES
Handling.V.Eating food*10 months to 3 years.v.3 to 6 years	1.225	1.001	1.468	ROPE
Handling.V.Interactions with toys*10 months to 3 years.v.3 to 6 years	0.683	0.525	0.841	ROPE
Handling.V.Interactions with familiar people*10 months to 3 years.v.3 to 6 years	0.702	0.584	0.838	ROPE
Handling.V.Interactions with unfamiliar people*10 months to 3 years.v.3 to 6 years	0.846	0.704	0.977	ROPE
In kennel towards people.V.Out of kennel towards people*10 months to 3 years.v.3 to 6 years	0.662	0.538	0.796	YES
In kennel towards people.V.Eating food*10 months to 3 years.v.3 to 6 years	1.544	1.28	1.811	YES
In kennel towards people.V.Interactions with toys*10 months to 3 years.v.3 to 6 years	0.86	0.68	1.057	ROPE
In kennel towards people.V.Interactions with familiar people*10 months to 3 years.v.3 to 6 years	0.884	0.742	1.023	ROPE
In kennel towards people.V.Interactions with unfamiliar people*10 months to 3 years.v.3 to 6 years	1.066	0.919	1.225	NULL
Out of kennel towards people.V.Eating food*10 months to 3 years.v.3 to 6 years	2.349	1.891	2.925	YES
Out of kennel towards people.V.Interactions with toys*10 months to 3 years.v.3 to 6 years	1.309	0.99	1.664	ROPE
Out of kennel towards people.V.Interactions with familiar people*10 months to 3 years.v.3 to 6 years	1.346	1.07	1.644	ROPE
Out of kennel towards people.V.Interactions with unfamiliar people*10 months to 3 years.v.3 to 6 years	1.622	1.312	1.965	YES
Eating food.V.Interactions with toys*10 months to 3 years.v.3 to 6 years	0.56	0.425	0.689	YES
Eating food.V.Interactions with familiar people*10 months to 3 years.v.3 to 6 years	0.576	0.468	0.687	YES
Eating food.V.Interactions with unfamiliar people*10 months to 3 years.v.3 to 6 years	0.694	0.572	0.823	ROPE
Interactions with toys.V.Interactions with familiar people*10 months to 3 years.v.3 to 6 years	1.038	0.804	1.291	ROPE
Interactions with toys.V.Interactions with unfamiliar people*10 months to 3 years.v.3 to 6 years	1.252	0.963	1.522	ROPE
Interactions with familiar people.V.Interactions with unfamiliar people*10 months to 3 years.v.3 to 6 years	1.211	1.008	1.41	ROPE
Handling.V.In kennel towards people*10 months to 3 years.v.Over 6 years	0.283	0.23	0.338	YES
Handling.V.Out of kennel towards people*10 months to 3 years.v.Over 6 years	0.291	0.22	0.367	YES
Handling.V.Eating food*10 months to 3 years.v.Over 6 years	1.106	0.887	1.35	ROPE
Handling.V.Interactions with toys*10 months to 3 years.v.Over 6 years	0.938	0.722	1.178	ROPE
Handling.V.Interactions with familiar people*10 months to 3 years.v.Over 6 years	0.417	0.33	0.5	YES
Handling.V.Interactions with unfamiliar people*10 months to 3 years.v.Over 6 years	0.306	0.243	0.364	YES
In kennel towards people.V.Out of kennel towards people*10 months to 3 years.v.Over 6 years	1.032	0.776	1.295	ROPE
In kennel towards people.V.Eating food*10 months to 3 years.v.Over 6 years	3.92	3.125	4.74	YES
In kennel towards people.V.Interactions with toys*10 months to 3 years.v.Over 6 years	3.323	2.576	4.135	YES
In kennel towards people.V.Interactions with familiar people*10 months to 3 years.v.Over 6 years	1.478	1.18	1.763	ROPE

In kennel towards people.V.Interactions with unfamiliar people*10 months to 3 years.v.Over 6 years	1.083	0.879	1.287	ROPE
Out of kennel towards people.V.Eating food*10 months to 3 years.v.Over 6 years	3.843	2.867	4.862	YES
Out of kennel towards people.V.Interactions with toys*10 months to 3 years.v.Over 6 years	3.258	2.318	4.22	YES
Out of kennel towards people.V.Interactions with familiar people*10 months to 3 years.v.Over 6 years	1.449	1.068	1.823	ROPE
Out of kennel towards people.V.Interactions with unfamiliar people*10 months to 3 years.v.Over 6 years	1.062	0.805	1.342	ROPE
Eating food.V.Interactions with toys*10 months to 3 years.v.Over 6 years	0.853	0.658	1.071	ROPE
Eating food.V.Interactions with familiar people*10 months to 3 years.v.Over 6 years	0.379	0.3	0.465	YES
Eating food.V.Interactions with unfamiliar people*10 months to 3 years.v.Over 6 years	0.278	0.222	0.336	YES
Interactions with toys.V.Interactions with familiar people*10 months to 3 years.v.Over 6 years	0.449	0.341	0.565	YES
Interactions with toys.V.Interactions with unfamiliar people*10 months to 3 years.v.Over 6 years	0.329	0.255	0.412	YES
Interactions with familiar people.V.Interactions with unfamiliar people*10 months to 3 years.v.Over 6 years	0.738	0.59	0.887	ROPE
Handling.V.In kennel towards people*3 to 6 years.v.Over 6 years	0.357	0.291	0.43	YES
Handling.V.Out of kennel towards people*3 to 6 years.v.Over 6 years	0.558	0.409	0.709	YES
Handling.V.Eating food*3 to 6 years.v.Over 6 years	0.907	0.717	1.106	ROPE
Handling.V.Interactions with toys*3 to 6 years.v.Over 6 years	1.384	1.043	1.753	ROPE
Handling.V.Interactions with familiar people*3 to 6 years.v.Over 6 years	0.597	0.469	0.727	YES
Handling.V.Interactions with unfamiliar people*3 to 6 years.v.Over 6 years	0.363	0.292	0.438	YES
In kennel towards people.V.Out of kennel towards people*3 to 6 years.v.Over 6 years	1.569	1.151	1.993	ROPE
In kennel towards people.V.Eating food*3 to 6 years.v.Over 6 years	2.55	2.009	3.069	YES
In kennel towards people.V.Interactions with toys*3 to 6 years.v.Over 6 years	3.892	2.959	4.921	YES
In kennel towards people.V.Interactions with familiar people*3 to 6 years.v.Over 6 years	1.678	1.357	2.054	YES
In kennel towards people.V.Interactions with unfamiliar people*3 to 6 years.v.Over 6 years	1.019	0.825	1.217	NULL
Out of kennel towards people.V.Eating food*3 to 6 years.v.Over 6 years	1.648	1.205	2.106	ROPE
Out of kennel towards people.V.Interactions with toys*3 to 6 years.v.Over 6 years	2.516	1.737	3.322	YES
Out of kennel towards people.V.Interactions with familiar people*3 to 6 years.v.Over 6 years	1.085	0.79	1.413	ROPE
Out of kennel towards people.V.Interactions with unfamiliar people*3 to 6 years.v.Over 6 years	0.659	0.489	0.84	ROPE
Eating food.V.Interactions with toys*3 to 6 years.v.Over 6 years	1.536	1.149	1.957	ROPE
Eating food.V.Interactions with familiar people*3 to 6 years.v.Over 6 years	0.662	0.523	0.824	ROPE
Eating food.V.Interactions with unfamiliar people*3 to 6 years.v.Over 6 years	0.402	0.317	0.486	YES
Interactions with toys.V.Interactions with familiar people*3 to 6 years.v.Over 6 years	0.436	0.332	0.564	YES
Interactions with toys.V.Interactions with unfamiliar people*3 to 6 years.v.Over 6 years	0.265	0.201	0.335	YES

Interactions with familiar people.V.Interactions with unfamiliar people*3 to 6 years.v.Over 6 years	0.612	0.481	0.743	YES
