# Supplementary material for: Aggressiveness as a latent personality trait of domestic dogs: Testing local independence and measurement invariance
Source: PLoS One. 2017 Aug 30;12(8):e0183595. doi: 10.1371/journal.pone.0183595 (PMC5576744; doi:10.1371/journal.pone.0183595)
Supplement: S4 Table — Mean and 95% highest density interval (HDI) estimates for all parameters from the Bayesian hierarchical logistic model assessing measurement invariance for contexts reflecting aggressiveness towards dogs. Differences between levels of categorical variables are indicated by ‘.v.’ in the parameter name; interactions are denoted with ‘*’ in the parameter name. The decision rule for each parameter is given except for those variables not interpreted inferentially: YES = 95% HDI falls completely outside the region of practical equivalence (ROPE); NULL = 95% HDI falls completely inside the ROPE; ROPE = 95% HDI partly covers the ROPE. (PDF) [file pone.0183595.s005.pdf]

**S4 Table. Bayesian hierarchical model parameter estimates for aggression towards dogs in different contexts.** Mean and 95% highest density interval (HDI) estimates for all parameters from the Bayesian hierarchical logistic model assessing measurement invariance for contexts reflecting aggressiveness towards dogs. Differences between levels of categorical variables are indicated by '.v.' in the parameter name; interactions are denoted with '*' in the parameter name. The decision rule for each parameter is given except for those variables not interpreted inferentially: YES = 95% HDI falls completely outside the region of practical equivalence (ROPE); NULL = 95% HDI falls completely inside the ROPE; ROPE = 95% HDI partly covers the ROPE.

| Parameter | Mean | HDI low | HDI high | Decision |
|---|---|---|---|---|
| Weight | 1.163 | 1.107 | 1.218 | Not interpreted |
| Total days | 2.232 | 2.125 | 2.342 | Not interpreted |
| London .v. Brands Hatch centre | 0.756 | 0.697 | 0.813 | Not interpreted |
| London .v. Old Windsor shelter | 0.941 | 0.886 | 0.996 | Not interpreted |
| Brands Hatch .v. Old Windsor shelter | 0.804 | 0.736 | 0.874 | Not interpreted |
| Not neutered .v. neutered on site | 2.013 | 1.884 | 2.147 | Not interpreted |
| Not neutered .v. neutered | 1.454 | 1.362 | 1.546 | Not interpreted |
| Neutered .v. neutered on site | 1.385 | 1.294 | 1.472 | Not interpreted |
| Gift.v.Return | 0.762 | 0.686 | 0.845 | Not interpreted |
| Gift.v.Stray | 1.227 | 1.157 | 1.295 | Not interpreted |
| Stray.v.return | 0.622 | 0.555 | 0.692 | Not interpreted |
| Females.v.males | 1.187 | 1.128 | 1.250 | Not interpreted |
| Interactions with female dogs.v.Interactions with male dogs | 0.699 | 0.661 | 0.737 | YES |
| Interactions with female dogs.v.In kennel towards dogs | 2.095 | 1.969 | 2.230 | YES |
| Interactions with female dogs.v.Out of kennel towards dogs | 1.306 | 1.234 | 1.380 | ROPE |
| Interactions with male dogs.V.In kennel towards dogs | 3.00 | 2.822 | 3.191 | YES |
| Interactions with male dogs.V.Out of kennel towards dogs | 1.306 | 1.234 | 1.380 | ROPE |
| In kennel towards dogs.V.Out of kennel towards dogs | 0.624 | 0.585 | 0.662 | YES |
| 4 to 10 months.v.10 months to 3 years | 0.571 | 0.527 | 0.616 | YES |
| 4 to 10 months.v.3 to 6 years | 0.513 | 0.471 | 0.555 | YES |
| 4 to 10 months.v.Over 6 years | 0.616 | 0.559 | 0.678 | YES |
| 10 months to 3 years.v.3 to 6 years | 0.899 | 0.850 | 0.956 | NULL |
| 10 months to 3 years.v.Over 6 years | 1.078 | 0.995 | 1.155 | NULL |
| 3 to 6 years.v.Over 6 years | 1.200 | 1.113 | 1.291 | ROPE |
| Interactions with female dogs.V.Interactions with male dogs*female.v.male | 1.542 | 1.400 | 1.704 | YES |
| Interactions with female dogs.V.In kennel towards dogs*female.v.male | 1.018 | 0.907 | 1.128 | NULL |
| Interactions with female dogs.V.Out of kennel towards dogs*female.v.male | 1.444 | 1.301 | 1.603 | YES |
| Interactions with male dogs.V.In kennel towards dogs*female.v.male | 0.661 | 0.590 | 0.732 | YES |

| | | | | |
|---|---|---|---|---|
| Interactions with male dogs.V.Out of kennel towards dogs*female.v.male | 0.938 | 0.838 | 1.029 | NULL |
| In kennel towards dogs.V.Out of kennel towards dogs*female.v.male | 1.420 | 1.269 | 1.587 | YES |
| Interactions with female dogs.V.Interactions with male dogs*4 to 10 months.v.10 months to 3 years | 1.198 | 1.003 | 1.391 | ROPE |
| Interactions with female dogs.V.In kennel towards dogs*4 to 10 months.v.10 months to 3 years | 0.984 | 0.808 | 1.171 | NULL |
| Interactions with female dogs.V.Out of kennel towards dogs*4 to 10 months.v.10 months to 3 years | 0.595 | 0.495 | 0.688 | YES |
| Interactions with male dogs.V.In kennel towards dogs*4 to 10 months.v.10 months to 3 years | 0.824 | 0.683 | 0.983 | ROPE |
| Interactions with male dogs.V.Out of kennel towards dogs*4 to 10 months.v.10 months to 3 years | 0.499 | 0.422 | 0.575 | YES |
| In kennel towards dogs.V.Out of kennel towards dogs*4 to 10 months.v.10 months to 3 years | 0.608 | 0.505 | 0.728 | YES |
| Interactions with female dogs.V.Interactions with male dogs*4 to 10 months.v.3 to 6 years | 1.202 | 1.015 | 1.412 | ROPE |
| Interactions with female dogs.V.In kennel towards dogs*4 to 10 months.v.3 to 6 years | 0.956 | 0.773 | 1.133 | ROPE |
| Interactions with female dogs.V.Out of kennel towards dogs*4 to 10 months.v.3 to 6 years | 0.263 | 0.22 | 0.307 | YES |
| Interactions with male dogs.V.In kennel towards dogs*4 to 10 months.v.3 to 6 years | 0.798 | 0.65 | 0.944 | ROPE |
| Interactions with male dogs.V.Out of kennel towards dogs*4 to 10 months.v.3 to 6 years | 0.22 | 0.185 | 0.256 | YES |
| In kennel towards dogs.V.Out of kennel towards dogs*4 to 10 months.v.3 to 6 years | 0.277 | 0.227 | 0.331 | YES |
| Interactions with female dogs.V.Interactions with male dogs*4 to 10 months.v.Over 6 years | 1.441 | 1.175 | 1.697 | ROPE |
| Interactions with female dogs.V.In kennel towards dogs*4 to 10 months.v.Over 6 years | 0.965 | 0.768 | 1.172 | ROPE |
| Interactions with female dogs.V.Out of kennel towards dogs*4 to 10 months.v.Over 6 years | 0.38 | 0.308 | 0.449 | YES |
| Interactions with male dogs.V.In kennel towards dogs*4 to 10 months.v.Over 6 years | 0.672 | 0.545 | 0.816 | ROPE |
| Interactions with male dogs.V.Out of kennel towards dogs*4 to 10 months.v.Over 6 years | 0.265 | 0.219 | 0.315 | YES |
| In kennel towards dogs.V.Out of kennel towards dogs*4 to 10 months.v.Over 6 years | 0.396 | 0.316 | 0.481 | YES |
| Interactions with female dogs.V.Interactions with male dogs*10 months to 3 years.v.3 to 6 years | 1.005 | 0.897 | 1.117 | NULL |
| Interactions with female dogs.V.In kennel towards dogs*10 months to 3 years.v.3 to 6 years | 0.973 | 0.85 | 1.09 | NULL |
| Interactions with female dogs.V.Out of kennel towards dogs*10 months to 3 years.v.3 to 6 years | 0.443 | 0.39 | 0.499 | YES |
| Interactions with male dogs.V.In kennel towards dogs*10 months to 3 years.v.3 to 6 years | 0.97 | 0.862 | 1.103 | NULL |
| Interactions with male dogs.V.Out of kennel towards dogs*10 months to 3 years.v.3 to 6 years | 0.442 | 0.389 | 0.496 | YES |
| In kennel towards dogs.V.Out of kennel towards dogs*10 months to 3 years.v.3 to 6 years | 0.456 | 0.396 | 0.516 | YES |
| Interactions with female dogs.V.Interactions with male dogs*10 months to 3 years.v.Over 6 years | 1.205 | 1.052 | 1.374 | ROPE |
| Interactions with female dogs.V.In kennel towards dogs*10 months to 3 years.v.Over 6 years | 0.982 | 0.829 | 1.131 | NULL |
| Interactions with female dogs.V.Out of kennel towards dogs*10 months to 3 years.v.Over 6 years | 0.639 | 0.544 | 0.734 | YES |

| | | | | |
|---|---|---|---|---|
| Interactions with male dogs.V.In kennel towards dogs*10 months to 3 years.v.Over 6 years | 0.817 | 0.696 | 0.945 | ROPE |
| Interactions with male dogs.V.Out of kennel towards dogs*10 months to 3 years.v.Over 6 years | 0.532 | 0.457 | 0.611 | YES |
| In kennel towards dogs.V.Out of kennel towards dogs*10 months to 3 years.v.Over 6 years | 0.653 | 0.545 | 0.751 | YES |
| Interactions with female dogs.V.Interactions with male dogs*3 to 6 years.v.Over 6 years | 1.201 | 1.043 | 1.365 | ROPE |
| Interactions with female dogs.V.In kennel towards dogs*3 to 6 years.v.Over 6 years | 1.011 | 0.862 | 1.182 | NULL |
| Interactions with female dogs.V.Out of kennel towards dogs*3 to 6 years.v.Over 6 years | 1.446 | 1.229 | 1.656 | ROPE |
| Interactions with male dogs.V.In kennel towards dogs*3 to 6 years.v.Over 6 years | 0.844 | 0.721 | 0.976 | ROPE |
| Interactions with male dogs.V.Out of kennel towards dogs*3 to 6 years.v.Over 6 years | 1.206 | 1.033 | 1.394 | ROPE |
| In kennel towards dogs.V.Out of kennel towards dogs*3 to 6 years.v.Over 6 years | 1.435 | 1.205 | 1.681 | ROPE |
